# *N*-(2-Methyl­phen­yl)-2-nitro­benzamide

**DOI:** 10.1107/S1600536808002298

**Published:** 2008-01-25

**Authors:** Aamer Saeed, Shahid Hussain, Michael Bolte

**Affiliations:** a Department of Chemistry, Quaid-i-Azam University, Islamabad, Pakistan; b Institut für Anorganische Chemie, J. W. Goethe-Universität Frankfurt, Max-von-Laue-Strasse 7, 60438 Frankfurt/Main, Germany

## Abstract

In the title compound, C_14_H_12_N_2_O_3_, the dihedral angle between the two aromatic rings is 41.48 (5)°. The nitro group is twisted by 24.7 (3)° out of the plane of the aromatic ring to which it is attached. The mol­ecules are connected by N—H⋯O hydrogen bonds into chains running along the *a* axis.

## Related literature

For related literature, see: Igawa *et al.* (1999 ▶); Jackson *et al.* (1994 ▶); Makino *et al.* (2001 ▶, 2003 ▶); Manley *et al.* (2002 ▶); Zhichkin *et al.* (2007 ▶); Capdeville *et al.* (2002 ▶); Ho *et al.* (2002 ▶).
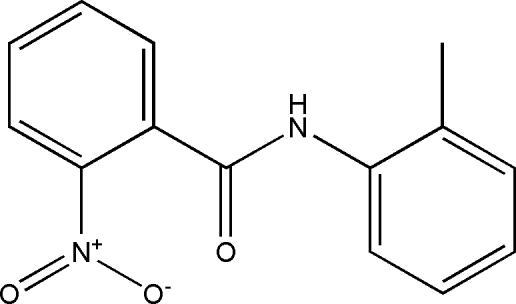


## Experimental

### 

#### Crystal data

C_14_H_12_N_2_O_3_
*M* *_r_* = 256.26
Orthorhombic, 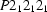

*a* = 7.8063 (10) Å
*b* = 12.2856 (11) Å
*c* = 13.1353 (13) Å
*V* = 1259.7 (2) Å^3^
*Z* = 4
Mo *K*α radiation
μ = 0.10 mm^−1^
*T* = 273 (2) K
0.35 × 0.14 × 0.13 mm

#### Data collection

Stoe IPDSII two-circle diffractometer
Absorption correction: none
4741 measured reflections
1364 independent reflections
1243 reflections with *I* > 2σ(*I*)
*R*_int_ = 0.036

#### Refinement

*R*[*F* ^2^ > 2σ(*F* ^2^)] = 0.031
*wR*(*F* ^2^) = 0.077
*S* = 1.02
1364 reflections
178 parameters
H atoms treated by a mixture of independent and constrained refinement
Δρ_max_ = 0.13 e Å^−3^
Δρ_min_ = −0.14 e Å^−3^

### 

Data collection: *X-AREA* (Stoe & Cie, 2001 ▶); cell refinement: *X-AREA*; data reduction: *X-AREA*; program(s) used to solve structure: *SHELXS97* (Sheldrick, 2008 ▶); program(s) used to refine structure: *SHELXL97* (Sheldrick, 2008 ▶); molecular graphics: *PLATON* (Spek, 2003 ▶); software used to prepare material for publication: *SHELXL97*.

## Supplementary Material

Crystal structure: contains datablocks I, global. DOI: 10.1107/S1600536808002298/at2536sup1.cif

Structure factors: contains datablocks I. DOI: 10.1107/S1600536808002298/at2536Isup2.hkl

Additional supplementary materials:  crystallographic information; 3D view; checkCIF report

## Figures and Tables

**Table 1:** Hydrogen-bond geometry (Å, °)

*D*—H⋯*A*	*D*—H	H⋯*A*	*D*⋯*A*	*D*—H⋯*A*
N1—H1⋯O1^i^	0.92 (3)	1.99 (3)	2.849 (2)	155 (2)

Symmetry code: (i) 
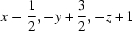
.

**Fig. 1.**
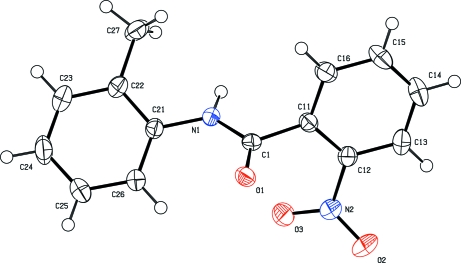
Molecular structure of title compound with displacement ellipsoids at the 50% probability level.

## supplementary crystallographic information

### Comment

The benzanilide core is present in compounds with such a wide range of biological activities that it has been called a privileged structure. Benzanilides serve as intermediates towards benzothiadiazin-4-ones (Makino *et al.*, 2003), quinazoline-2,4-diones (Makino *et al.*, 2001) and benzodiazepine-2,5-diones (Ho *et al.*, 2002) and 110 kinase inhibitors 2,3-disubstituted 3*H*-quinazoline-4-ones (Zhichkin *et al.*, 2007). Benzanilides have established their efficacy as centroid elements of ligands that bind to a wide variety of receptor types. Thus benzanilides containing aminoalkyl groups originally designed as a peptidomimetic, have been incorporated in an Arg-Gly-Asp cyclic peptide yielding a high affinity GPIIb/IIIa ligand (Jackson *et al.*, 1994). Imatinib is an ATP-site binding kinase inhibitor and platelet-derived growth factor receptor kinases (Capdeville *et al.*, 2002). Pyridylmethyl containing benzanilide are vascular endothelial growth factor receptor and tyrosine kinase inhibitor (Manley *et al.*, 2002). Furthermore, benzamides have been reported to have activities as acetyl-CoA carboxylase and farnesyl transferase inhibitors (Igawa *et al.*, 1999)

Geometric parameters of the title compound are in the usual ranges. The dihedral angle between the two aromatic rings is 41.48 (5)°. The nitro group is twisted by 24.7 (3)° out of the plane of the aromatic ring to which it is attached. The molecules crystallize in chains running along the *a* axis. The molecules in a chain are connected by N—H···O hydrogen bonds.

### Refinement

Due to the absence of any anomalous scatterer, Friedel pairs were merged prior to refinement and the absolute structure was arbitrarily set. All H atoms were found in a difference map, but those bonded to C were geometrically positioned and refined with fixed individual displacement parameters [*U*_iso_(H) = 1.2or 1.5 *U*_eq_(C)] using a riding model with C—H = 0.95 Å. The amino H atom was freely refined.

### Crystal data

**Table:**

C_14_H_12_N_2_O_3_	*F*_000_ = 536
*M**_r_* = 256.26	*D*_x_ = 1.351 Mg m^−^^3^
Orthorhombic, *P*2_1_2_1_2_1_	Mo *K*α radiation λ = 0.71073 Å
Hall symbol: P 2ac 2ab	Cell parameters from 3989 reflections
*a* = 7.8063 (10) Å	θ = 3.6–25.7º
*b* = 12.2856 (11) Å	µ = 0.10 mm^−^^1^
*c* = 13.1353 (13) Å	*T* = 273 (2) K
*V* = 1259.7 (2) Å^3^	Needle, light brown
*Z* = 4	0.35 × 0.14 × 0.13 mm

### Data collection

**Table:**

Stoe IPDSII two-circle diffractometer	1243 reflections with *I* > 2σ(*I*)
Radiation source: fine-focus sealed tube	*R*_int_ = 0.036
Monochromator: graphite	θ_max_ = 25.5º
*T* = 273(2) K	θ_min_ = 3.5º
ω scans	*h* = −8→9
Absorption correction: none	*k* = −14→12
4741 measured reflections	*l* = −15→15
1364 independent reflections	

### Refinement

**Table:**

Refinement on *F*^2^	Hydrogen site location: inferred from neighbouring sites
Least-squares matrix: full	H atoms treated by a mixture of independent and constrained refinement
*R*[*F*^2^ > 2σ(*F*^2^)] = 0.031	*w* = 1/[σ^2^(*F*_o_^2^) + (0.0533*P*)^2^ + 0.0696*P*] where *P* = (*F*_o_^2^ + 2*F*_c_^2^)/3
*wR*(*F*^2^) = 0.078	(Δ/σ)_max_ < 0.001
*S* = 1.02	Δρ_max_ = 0.13 e Å^−^^3^
1364 reflections	Δρ_min_ = −0.14 e Å^−^^3^
178 parameters	Extinction correction: SHELXL97 (Sheldrick, 1997), Fc^*^=kFc[1+0.001xFc^2^λ^3^/sin(2θ)]^-1/4^
Primary atom site location: structure-invariant direct methods	Extinction coefficient: 0.017 (3)
Secondary atom site location: difference Fourier map	

### Special details

**Table:**

Geometry. All e.s.d.'s (except the e.s.d. in the dihedral angle between two l.s. planes) are estimated using the full covariance matrix. The cell e.s.d.'s are taken into account individually in the estimation of e.s.d.'s in distances, angles and torsion angles; correlations between e.s.d.'s in cell parameters are only used when they are defined by crystal symmetry. An approximate (isotropic) treatment of cell e.s.d.'s is used for estimating e.s.d.'s involving l.s. planes.
Refinement. Refinement of *F*^2^ against ALL reflections. The weighted *R*-factor *wR* and goodness of fit *S* are based on *F*^2^, conventional *R*-factors *R* are based on *F*, with *F* set to zero for negative *F*^2^. The threshold expression of *F*^2^ > σ(*F*^2^) is used only for calculating *R*-factors(gt) *etc*. and is not relevant to the choice of reflections for refinement. *R*-factors based on *F*^2^ are statistically about twice as large as those based on *F*, and *R*- factors based on ALL data will be even larger.

### Fractional atomic coordinates and isotropic or equivalent isotropic displacement parameters (Å^2^)

**Table:**

	*x*	*y*	*z*	*U*_iso_*/*U*_eq_	
N1	0.6342 (2)	0.68690 (13)	0.54004 (11)	0.0240 (4)	
H1	0.553 (3)	0.726 (2)	0.5746 (17)	0.040 (7)*	
C1	0.7172 (2)	0.73801 (15)	0.46407 (13)	0.0227 (4)	
O1	0.82960 (19)	0.69697 (10)	0.41024 (10)	0.0277 (3)	
N2	0.5272 (2)	0.82075 (14)	0.28700 (12)	0.0302 (4)	
O2	0.5146 (3)	0.85091 (14)	0.19817 (11)	0.0495 (5)	
O3	0.4810 (2)	0.73064 (12)	0.31740 (11)	0.0388 (4)	
C11	0.6745 (2)	0.85748 (16)	0.45063 (13)	0.0239 (4)	
C12	0.5996 (3)	0.89811 (16)	0.36185 (14)	0.0251 (4)	
C13	0.5832 (3)	1.00790 (17)	0.34180 (16)	0.0341 (5)	
H13	0.5322	1.0317	0.2818	0.041*	
C14	0.6439 (3)	1.08187 (18)	0.41256 (18)	0.0405 (5)	
H14	0.6340	1.1562	0.4004	0.049*	
C15	0.7190 (3)	1.04525 (17)	0.50114 (18)	0.0383 (5)	
H15	0.7598	1.0952	0.5485	0.046*	
C16	0.7347 (3)	0.93358 (17)	0.52071 (16)	0.0321 (5)	
H16	0.7856	0.9101	0.5808	0.038*	
C21	0.6503 (3)	0.57434 (16)	0.56687 (14)	0.0246 (4)	
C22	0.6130 (3)	0.54470 (17)	0.66744 (14)	0.0284 (4)	
C23	0.6223 (3)	0.43408 (19)	0.69291 (17)	0.0384 (5)	
H23	0.5968	0.4124	0.7590	0.046*	
C24	0.6687 (4)	0.35605 (18)	0.62128 (18)	0.0417 (6)	
H24	0.6756	0.2831	0.6399	0.050*	
C25	0.7045 (3)	0.38710 (17)	0.52281 (17)	0.0392 (5)	
H25	0.7353	0.3349	0.4749	0.047*	
C26	0.6949 (3)	0.49615 (17)	0.49469 (15)	0.0316 (5)	
H26	0.7181	0.5168	0.4280	0.038*	
C27	0.5619 (3)	0.6283 (2)	0.74710 (14)	0.0365 (5)	
H27A	0.6471	0.6846	0.7501	0.055*	
H27B	0.5531	0.5935	0.8124	0.055*	
H27C	0.4533	0.6595	0.7292	0.055*	

### Atomic displacement parameters (Å^2^)

**Table:**

	*U*^11^	*U*^22^	*U*^33^	*U*^12^	*U*^13^	*U*^23^
N1	0.0313 (9)	0.0204 (8)	0.0201 (8)	0.0029 (7)	0.0025 (7)	0.0010 (6)
C1	0.0261 (10)	0.0223 (9)	0.0198 (8)	−0.0011 (8)	−0.0039 (7)	−0.0026 (7)
O1	0.0326 (8)	0.0226 (7)	0.0278 (7)	−0.0013 (6)	0.0060 (6)	−0.0034 (5)
N2	0.0338 (9)	0.0316 (10)	0.0251 (8)	0.0001 (8)	−0.0025 (7)	0.0009 (7)
O2	0.0736 (13)	0.0524 (10)	0.0226 (7)	−0.0003 (10)	−0.0107 (8)	0.0060 (7)
O3	0.0504 (10)	0.0302 (7)	0.0358 (7)	−0.0138 (7)	−0.0059 (7)	−0.0001 (6)
C11	0.0259 (10)	0.0206 (9)	0.0252 (9)	0.0001 (8)	0.0029 (8)	−0.0013 (7)
C12	0.0259 (10)	0.0244 (10)	0.0250 (9)	−0.0034 (8)	0.0018 (8)	0.0009 (7)
C13	0.0388 (12)	0.0262 (11)	0.0373 (10)	0.0009 (9)	0.0015 (10)	0.0096 (9)
C14	0.0439 (13)	0.0210 (10)	0.0565 (14)	0.0021 (10)	−0.0018 (11)	0.0034 (10)
C15	0.0425 (13)	0.0233 (10)	0.0492 (13)	−0.0015 (10)	−0.0036 (11)	−0.0101 (9)
C16	0.0377 (12)	0.0271 (10)	0.0314 (10)	0.0034 (9)	−0.0054 (9)	−0.0050 (8)
C21	0.0274 (10)	0.0224 (9)	0.0242 (8)	0.0002 (8)	−0.0025 (8)	0.0036 (7)
C22	0.0311 (10)	0.0300 (10)	0.0241 (9)	0.0019 (9)	−0.0024 (8)	0.0053 (8)
C23	0.0465 (13)	0.0357 (12)	0.0330 (10)	0.0005 (10)	−0.0003 (10)	0.0134 (9)
C24	0.0532 (14)	0.0230 (10)	0.0490 (12)	0.0001 (11)	0.0022 (12)	0.0116 (10)
C25	0.0522 (14)	0.0231 (10)	0.0422 (11)	−0.0016 (10)	0.0020 (11)	−0.0017 (9)
C26	0.0439 (13)	0.0230 (9)	0.0278 (10)	−0.0016 (9)	0.0016 (9)	0.0020 (8)
C27	0.0451 (13)	0.0436 (13)	0.0209 (9)	0.0077 (11)	0.0032 (9)	0.0031 (9)

### Geometric parameters (Å, °)

**Table:**

N1—C1	1.345 (2)	C15—H15	0.9300
N1—C21	1.433 (2)	C16—H16	0.9300
N1—H1	0.92 (3)	C21—C26	1.394 (3)
C1—O1	1.234 (2)	C21—C22	1.401 (3)
C1—C11	1.515 (3)	C22—C23	1.401 (3)
N2—O2	1.228 (2)	C22—C27	1.519 (3)
N2—O3	1.231 (2)	C23—C24	1.391 (3)
N2—C12	1.480 (3)	C23—H23	0.9300
C11—C16	1.393 (3)	C24—C25	1.377 (3)
C11—C12	1.397 (3)	C24—H24	0.9300
C12—C13	1.380 (3)	C25—C26	1.392 (3)
C13—C14	1.384 (3)	C25—H25	0.9300
C13—H13	0.9300	C26—H26	0.9300
C14—C15	1.379 (3)	C27—H27A	0.9600
C14—H14	0.9300	C27—H27B	0.9600
C15—C16	1.401 (3)	C27—H27C	0.9600
			
C1—N1—C21	126.20 (17)	C15—C16—H16	119.8
C1—N1—H1	117.5 (15)	C26—C21—C22	120.94 (18)
C21—N1—H1	116.1 (16)	C26—C21—N1	121.32 (16)
O1—C1—N1	125.21 (18)	C22—C21—N1	117.68 (17)
O1—C1—C11	119.02 (17)	C21—C22—C23	117.80 (19)
N1—C1—C11	115.63 (16)	C21—C22—C27	121.89 (18)
O2—N2—O3	123.79 (18)	C23—C22—C27	120.30 (18)
O2—N2—C12	117.91 (17)	C24—C23—C22	121.35 (19)
O3—N2—C12	118.30 (16)	C24—C23—H23	119.3
C16—C11—C12	116.93 (18)	C22—C23—H23	119.3
C16—C11—C1	119.92 (16)	C25—C24—C23	119.8 (2)
C12—C11—C1	122.37 (16)	C25—C24—H24	120.1
C13—C12—C11	123.19 (18)	C23—C24—H24	120.1
C13—C12—N2	117.73 (18)	C24—C25—C26	120.3 (2)
C11—C12—N2	119.03 (17)	C24—C25—H25	119.8
C12—C13—C14	118.8 (2)	C26—C25—H25	119.8
C12—C13—H13	120.6	C25—C26—C21	119.76 (19)
C14—C13—H13	120.6	C25—C26—H26	120.1
C15—C14—C13	119.9 (2)	C21—C26—H26	120.1
C15—C14—H14	120.1	C22—C27—H27A	109.5
C13—C14—H14	120.1	C22—C27—H27B	109.5
C14—C15—C16	120.8 (2)	H27A—C27—H27B	109.5
C14—C15—H15	119.6	C22—C27—H27C	109.5
C16—C15—H15	119.6	H27A—C27—H27C	109.5
C11—C16—C15	120.4 (2)	H27B—C27—H27C	109.5
C11—C16—H16	119.8		
			
C21—N1—C1—O1	−5.4 (3)	C13—C14—C15—C16	−0.1 (4)
C21—N1—C1—C11	178.96 (17)	C12—C11—C16—C15	0.0 (3)
O1—C1—C11—C16	−103.0 (2)	C1—C11—C16—C15	170.1 (2)
N1—C1—C11—C16	72.9 (2)	C14—C15—C16—C11	0.1 (4)
O1—C1—C11—C12	66.5 (3)	C1—N1—C21—C26	−27.7 (3)
N1—C1—C11—C12	−117.6 (2)	C1—N1—C21—C22	154.78 (19)
C16—C11—C12—C13	−0.2 (3)	C26—C21—C22—C23	0.1 (3)
C1—C11—C12—C13	−170.0 (2)	N1—C21—C22—C23	177.60 (19)
C16—C11—C12—N2	−177.46 (18)	C26—C21—C22—C27	−179.2 (2)
C1—C11—C12—N2	12.7 (3)	N1—C21—C22—C27	−1.7 (3)
O2—N2—C12—C13	26.3 (3)	C21—C22—C23—C24	0.7 (3)
O3—N2—C12—C13	−152.8 (2)	C27—C22—C23—C24	−180.0 (2)
O2—N2—C12—C11	−156.3 (2)	C22—C23—C24—C25	−0.8 (4)
O3—N2—C12—C11	24.7 (3)	C23—C24—C25—C26	0.2 (4)
C11—C12—C13—C14	0.2 (3)	C24—C25—C26—C21	0.6 (4)
N2—C12—C13—C14	177.49 (19)	C22—C21—C26—C25	−0.7 (3)
C12—C13—C14—C15	0.0 (3)	N1—C21—C26—C25	−178.1 (2)

### Hydrogen-bond geometry (Å, °)

**Table:**

*D*—H···*A*	*D*—H	H···*A*	*D*···*A*	*D*—H···*A*
N1—H1···O1^i^	0.92 (3)	1.99 (3)	2.849 (2)	155 (2)

Symmetry codes: (i) *x*−1/2, −*y*+3/2, −*z*+1.
